# Remission of polycystic ovary syndrome based on the recovery of ovarian morphology identified by three-dimensional ultrasonography: a retrospective study

**DOI:** 10.3389/fendo.2026.1877873

**Published:** 2026-07-30

**Authors:** Keying Su, Yujie Zhao, Huihui Chai, Meili Cai, Diliqingna Dilimulati, Yuqin Zhang, Xiaowen Shao, Shen Qu, Chang Liu, Manna Zhang

**Affiliations:** 1 Department of Laboratory Medicine, Shanghai Tenth People’s Hospital, School of Medicine, Tongji University, Shanghai, China; 2 Department of General Practice, Shanghai Tenth People’s Hospital, School of Medicine, Tongji University, Shanghai, China; 3 Department of Medical Ultrasound, Shanghai Tenth People’s Hospital, School of Medicine, Tongji University, Shanghai, China; 4 Department of Endocrinology and Metabolism, Shanghai Tenth People’s Hospital, School of Medicine, Tongji University, Shanghai, China; 5 Department of Obstetrics and Gynecology, Shanghai Tenth People’s Hospital, School of Medicine, Tongji University, Shanghai, China

**Keywords:** metabolic disorder, polycystic ovarian morphology, polycystic ovary syndrome, remission, three-dimensional ultrasonography

## Abstract

**Background:**

Polycystic ovary syndrome (PCOS) is a prevalent chronic disease, yet the concept of PCOS remission and its predictors remain poorly understood.

**Objectives:**

This study investigated PCOS remission rates via three-dimensional (3D) ultrasonography assessment of ovarian morphological normalization.

**Methods:**

This retrospective study included 1379 reproductive-aged PCOS women from Shanghai Tenth People’s Hospital (Aug. 2019–Dec. 2025). After medication initiation, anthropometric, biochemical, and reproductive data (both baseline and post-treatment) were collected quarterly, while 3D ultrasonography was used to evaluate polycystic morphology during follow-up.

**Results:**

Among the 468 eligible participants, 55 (11.75%) achieved clinical remission of PCOS, defined as restoration of regular menses, normalization of serum testosterone levels, and recovery of normal ovarian morphology. The mean time to remission was 12.76 ± 11.77 months. Patients in the clinical remission group were frequently prescribed insulin-sensitizing and weight-loss agents, including metformin, glucagon-like peptide-1 receptor agonists (GLP-1 RAs), sodium-glucose linked transporter 2 inhibitors (SGLT-2is), and thiazolidinediones (TZDs). Following treatment, the remission group exhibited significant increases in menstrual cycle frequency and estradiol (E2), together with significant reductions in body mass index (BMI), waist circumference (WC), waist-to-hip ratio (WHR), alanine aminotransferase (ALT), aspartate aminotransferase (AST), γ-glutamyl transpeptidase (γ-GT), total cholesterol (TC), low-density lipoprotein cholesterol (LDL-c), uric acid (UA), total testosterone (TT), androstenedione, and anti-Müllerian hormone (AMH) compared with baseline. Significant decreases in bilateral ovarian volume and follicle number were also observed. After adjustment for multiple confounding factors, multivariate Cox proportional hazards regression revealed that BMI, ALT, and LDL-c retained strong independent predictive value for PCOS remission.

**Conclusion:**

Our study demonstrated that 11.75% of women with PCOS achieved complete clinical remission following targeted metabolic pharmacotherapy, as evidenced by restored ovarian morphology, normalized testosterone concentrations, and regular menstrual cycles.

**Clinical Trial Registration:**

http://www.chictr.org.cn, identifier: NCT05063383.

## Introduction

Polycystic ovary syndrome (PCOS) is a well-known prevalent endocrine disease characterized by hyperandrogenism, ovulation dysfunction, and polycystic ovarian morphology (PCOM), affecting women of reproductive age. It is studied that 10-15% of all women (1) or 5-20% of women in reproductive age (2) suffering from PCOS. PCOS is linked to metabolic complications like hyperinsulinism and insulin resistance (3), which raises cardiovascular risk factors and increases the prevalence of type 2 diabetes mellitus (T2DM), leading to a higher likelihood of cardiovascular disease and metabolic syndrome (4, 5). Thus, PCOS is defined as a metabolic disease that integrates endocrine and reproductive functions in recent studies (6).

Although PCOS is a chronic disorder, the concept of PCOS remission remains controversial, and few studies have explored the issue of remission in patients with PCOS. Complete remission of PCOS can be indicated by regular menstrual cycles or natural pregnancies in patients who have undergone surgeries such as laparoscopic sleeve gastrectomy or gastric bypass (7). SNS Samarasinghe et al. have reported that vertical sleeve gastrectomy was associated with a significantly increased rate of spontaneous ovulatory events, which biologically confirmed the regularization of menstrual cycles (8). However, the previous studies only exhibited the regularization of menstrual cycle in patients with PCOS; there was no data available on either the normalization of testosterone levels or the remission of PCOM, the latter being the main clinical manifestation, accounting for approximately 90% of patients with PCOS (96.7%, Kumarapeli et al., 92%, Zhang et al.) (9, 10). Notably, resolution of PCOM serves as a core marker of pathological improvement in PCOS and is pivotal to enhancing fertility outcomes and mitigating long-term disease-related risks, while also being critical to avoiding “symptomatic-only treatment”.

Uniform cutoffs for defining physiological ovarian morphology have not been universally established. In light of advances in high-resolution transvaginal ultrasonography (probe frequency ≥8 MHz), the 2018 and 2023 international PCOS guidelines have revised the diagnostic thresholds for polycystic ovarian morphology (PCOM). According to these updated criteria, PCOM is defined as an antral follicle count (AFC; 2–9 mm follicles) ≥20 per ovary or an ovarian volume ≥10 mL (11, 12). Building on these consensus criteria, we put forward a preliminary framework for ovarian morphological normalization. Accumulating evidence highlights three-dimensional (3D) ultrasonography offers significant advantages over two-dimensional (2D) methods in diagnosing PCOM. While 3D scanning requires dedicated hardware and standardized sonographer training, this modality achieves superior intra- and inter-observer reproducibility alongside streamlined quantitative antral follicle quantification (13–16). Its integrated automated follicle segmentation algorithm enables precise detection of small or overlapping follicles that are often indistinct on 2D scans (17). A major merit of 3D ultrasonography is its capability to optimize PCOS phenotypic diagnosis and inform personalized fertility counseling, with unique potential for longitudinal quantification of dynamic PCOM regression during serial follow-up of women with PCOS.

This study employed 3D ultrasonography to longitudinally assess ovarian morphological restoration, in conjunction with concurrent evaluations of menstrual cycle normalization and the resolution of hyperandrogenism in patients with PCOS. The study aspires to highlight the potential for achieving the regression of PCOM after pharmacological interventions, which carries important clinical implications and values for the management of PCOS.

## Methods

### Participants

**Figure 1:**
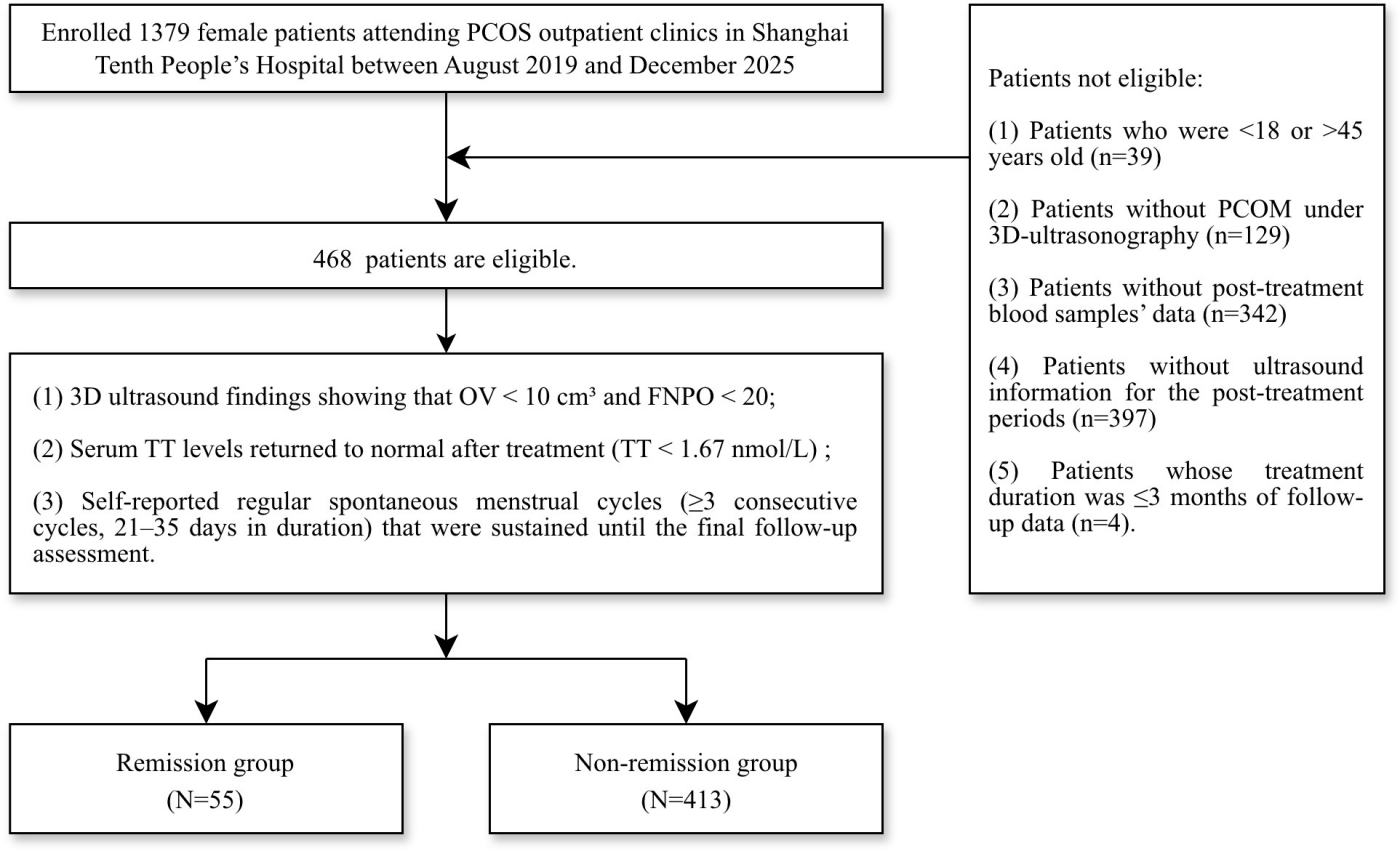
Flow diagram for screening and classification of patients with PCOS according to remission status.

This study adopted a retrospective cohort design to analyze medical data obtained from the PCOS outpatient clinics of Shanghai Tenth People’s Hospital (Clinical Trial Registration Number: NCT05063383). The study cohort consisted of women aged 18–45 years who were diagnosed with PCOS according to the 2003 Rotterdam criteria between August 2019 and December 2025 (18). The main clinical manifestations of the patients included menstrual dysfunction, infertility, acne, and hirsutism. A total of 468 patients were ultimately included in the data analysis (**Figure 1**). The exclusion criteria are as follows: (1) Age < 18 years or > 45 years; (2) Other endocrine disorders causing hyperandrogenism or ovulatory dysfunction (e.g., congenital adrenal hyperplasia, pituitary prolactinoma, thyroid dysfunction, Cushing’s syndrome); (3) Type 1/2 diabetes, severe dyslipidemia, or history of acute/chronic pancreatitis; (4) A history of bariatric surgery performed within 12 months prior to the study; (5) A personal or family history of medullary thyroid carcinoma, multiple endocrine neoplasia (MEN) type 2A or 2B, or any malignant tumor diagnosed at any time; (6) Severe organic diseases involving vital organs, including the heart, liver, kidneys; (7) A history of mental illness, drug or alcohol abuse, or poor compliance; (8) Use of medications known to affect insulin sensitivity (e.g., thiazolidinediones, metformin, acarbose, sodium-glucose cotransporter 2 inhibitors, glucagon-like peptide-1 receptor agonists) or PCOS-related hormonal treatments (e.g., oral contraceptives, gonadotropins, anti-androgens) within the preceding 3 months. According to the research guidelines of the Ethics Committee of Shanghai Tenth People’s Hospital, all patients participating in this study signed consent forms. The ethical approval number was SHSY-IEC-4.1/21-227/01.

### Data Collection and Follow-up

Patients’ weight, height, waist circumference (WC), and hip circumference (HC) were measured using calibrated equipment without shoes and heavy clothing or belongings. Body weight was measured in kilograms (kg) at each follow-up visit. Body mass index (BMI, kg/m²) was derived from weight (kg) divided by the square of height (m²), with results rounded to two decimal places. WC and HC were measured in centimeters. The waist-hip ratio (WHR) was computed as WC divided by HC.

Blood samples were collected and analyzed by professional laboratory personnel according to standardized clinical protocols. Venous blood samples were collected in the morning after a minimum of 10 hours of overnight fasting by trained personnel using standardized laboratory procedures. Fasting plasma glucose (FPG), fasting insulin (FINS), glycosylated hemoglobin A1c (HbA1c), alanine aminotransferase (ALT), aspartate aminotransferase (AST), total cholesterol (TC), triglyceride (TG), low-density lipoprotein cholesterol (LDL-c), high-density lipoprotein cholesterol (HDL-c), γ-glutamyl transpeptidase (γ-GT), creatinine (Cr), uric acid (UA), blood urea nitrogen (BUN), and estimated glomerular filtration rate (eGFR) were measured. Sex hormones and reproductive hormones, including luteinizing hormone (LH), follicle-stimulating hormone (FSH), total testosterone (TT), free testosterone (FT), prolactin (PRL), estradiol (E_2_), androstenedione (AD), dehydroepiandrosterone sulfate (DHEAS), sex hormone-binding globulin (SHBG), and anti-Müllerian hormone (AMH) were measured. The LH-FSH ratio was calculated by dividing LH (IU/L) by FSH (IU/L). Homeostasis model assessment of insulin resistance (HOMA-IR) was calculated according to the following equation: [FPG (mmol/L) × FINS (mU/L)]/22.5 (19).

To be specific, serum concentrations of sex hormones (LH, FSH, TT, FT, PRL, E2, AD, DHEA, SHBG and AMH) were determined by electrochemiluminescence immunoassay (ECLIA) using commercial Roche diagnostic kits on a Roche chemiluminescent immune-analyzer. All tests were performed according to the manufacturer’s instructions and laboratory standard operating procedures. Multi-point calibration with matched calibrators and dual-level internal quality controls were included in each analytical run. The Westgard multi-rule was applied for internal quality control, and the laboratory regularly participated in external quality assessment programs with all results within acceptable limits, guaranteeing the accuracy and reliability of measurements.

Participants’ medication strategy was systematically documented by well-trained researchers, including details such as medication names, dosages, frequency, start dates and subsequent adjustments. The self-reported length of menstrual cycle and the number of menstrual cycles were collected. Serum levels of sex hormones were measured in the morning after 10 hours of overnight fasting by trained personnel using standardized laboratory procedures. These data were followed up in our department by endocrinologist and nurses every 3 months (± 2 weeks) from medication initiation until the final follow-up cutoff date of December 2025.

### Ultrasound examination

All ultrasound examinations were performed by trained professional sonographers. Two high-end gynecological ultrasound systems were utilized for parallel data acquisition, including the GE Voluson E10 equipped with a transvaginal three-dimensional volume probe (RIC 8–9 D). The scanning approach (transvaginal or transrectal) was flexibly selected based on individual patient characteristics. To minimize equipment-related discrepancies, imaging parameters were standardized prior to acquisition. Following visualization of the maximum ovarian longitudinal section, 3D volume acquisition was initiated. The region of interest was tightly conformed to the ovarian boundaries with a minimal tissue margin, utilizing a standardized acquisition angle (90°–120°). Raw 3D datasets containing full grayscale information were stored losslessly in DICOM format to ensure platform-independent analysis. Virtual Organ Computer-Aided Analysis (VOCAL) technology was employed to delineate the ovarian capsule boundaries, reconstruct three-dimensional models, and calculate ovarian volumes in cubic centimeters (cm³). Additionally, the SonoAVC (automatic follicle volume and count) intelligent algorithm was enabled for the automated identification, segmentation, and counting of antral follicles. All data were collected exclusively by three-dimensional (3D) ultrasonography at baseline and during the entire follow-up period, allowing for quantification of ovarian volume (OV, cm³) and follicular number per ovary (FNPO).

### Therapeutic strategy

The therapeutic regimens for all patients in both groups were determined based on the following principles: All patients received health education focused on lifestyle interventions. Body mass index (BMI) was categorized according to the Chinese guideline as underweight (BMI < 18.5 kg/m²), normal weight (18.5 kg/m² ≤ BMI < 24.0 kg/m²), overweight (24.0 kg/m² ≤ BMI < 28.0 kg/m²), and obese (BMI ≥ 28.0 kg/m²) (20). Specifically, obese patients were administered glucagon-like peptide-1 receptor agonists (GLP-1RAs) monotherapy or a combination of GLP-1RAs and metformin; overweight patients were prescribed metformin monotherapy or a combination of metformin and sodium-glucose cotransporter 2 inhibitors (SGLT2is); patients with normal BMI received metformin monotherapy or a combination of metformin and pioglitazone; in normal-weight and overweight patients, if metformin therapy was ineffective or its adverse effects were intolerable, the treatment regimen was adjusted to SGLT2is; patients with BMI < 18.5 kg/m² were treated with pioglitazone monotherapy. Additionally, if patients had concurrent hyperlipidemia and met the recommended timing for initiating lipid-lowering therapy, statin or fibrate therapy was administered for targeted treatment (21). Given that prior literature has confirmed that agents for metabolic control confer definitive therapeutic benefits in PCOS patients without insulin resistance (22, 23), insulin resistance was not used as a criterion for selecting these medications in the regimens employed in the present study. Furthermore, hepatic and renal function, lipid profiles, and muscle enzyme levels were monitored every three months to ensure the safety of medication administration.

### Definition of remission

Patients with polycystic ovary syndrome (PCOS) were required to meet all three of the following criteria simultaneously, (1): 3D ultrasound findings showing that OV < 10 cm³ and FNPO < 20 (9, 11, 12, 15); (2) Serum TT levels returned to normal after treatment (TT < 1.67 nmol/L); (3) Self-reported regular spontaneous menstrual cycles (≥3 consecutive cycles, 21–35 days in duration) that were maintained through the final follow-up visit in the absence of any pharmacotherapy.

### Sample size

According to previous studies, among women with PCOS who have a BMI ≥ 27.5 kg/m² and waist circumference ≥ 85 cm, the remission rate—defined as achieving 6 consecutive regular menstrual cycles or spontaneous pregnancy—following treatment with oral medications (metformin and an oral contraceptive) is approximately 15% (7). Assuming a remission rate of 20%, a 95% confidence level, and a 5% margin of error, the minimum required sample size was 246, as calculated using the online population-proportion sample-size calculator available at https://select-statistics.co.uk/calculators/sample-size-calculator-population-proportion/.

### Statistical analysis

All statistical analyses were performed using IBM SPSS Statistics (version 27.0; IBM Corp., Armonk, NY, USA). Continuous variables were presented as mean ± standard deviation (SD) or median (interquartile range) based on their distribution. For baseline continuous variables, differences between the remission and non-remission groups were assessed using independent samples t-tests for normally distributed data; otherwise, the Mann-Whitney U test was employed. Within the remission group, changes in clinical parameters before and after treatment were evaluated using paired samples t-tests for normally distributed continuous variables, or Wilcoxon signed-rank tests for non-normally distributed variables. Treatment strategies and medication protocols were summarized, with results reported as counts and percentages. Cox proportional hazards regression models were applied to conduct univariate and multivariate analyses for identifying independent predictors of PCOS remission. Variables yielding a marginal association with PCOS remission (P < 0.05) in the univariate analysis or clinically relevant factors were subsequently incorporated into the multivariate Cox model via forward stepwise selection. Variance inflation factor (VIF) was calculated to evaluate multicollinearity among candidate variables; all variables retained in the final regression model exhibited a VIF < 5, confirming the absence of significant multicollinearity. The results were reported as hazard ratios (HRs) alongside their corresponding 95% confidence intervals (CIs). All statistical tests were two-tailed, and a P-value < 0.050 was defined as the threshold for statistical significance.

## Results

### Comparison of baseline characteristics in remission group and non-remission group

**Figure 1** presented a flowchart illustrating the patient selection process, which included 55 participants in the remission group and 413 in the non-remission group. At baseline, age, HbA1c levels, HOMA-IR, menstrual cycle numbers per year, liver and renal function parameters were comparable between the remission and non-remission groups. However, patients in the remission group had significantly higher WHR, TC, LDL-c, and FSH levels, along with lower LH/FSH, FT, and AMH levels, than those in the non-remission group (P < 0.050). The other sex hormone profiles and ovarian characteristics were comparable between the two groups (**Table 1**).

**Table 1:** Comparison of baseline demographic, serological, and ultrasonographic characteristics.

Items	Remission group (n=55)	Non-remission group (n=413)	P values
Age (years)	27.60 ± 4.48	27.22 ± 5.02	0.596
BMI (kg/m^2^)	26.00 ± 5.65	24.69 ± 5.19	0.082
WC (cm)	86.95 ± 12.43	83.11 ± 13.73	0.053
WHR	0.87 ± 0.08	0.80 ± 0.13	<0.001***
Menstrual cycles (no./year)	7.11 ± 3.71	7.99 ± 3.43	0.088
HbA1c (%)	5.52 ± 0.72	5.52 ± 0.49	0.935
HOMA-IR	4.08 ± 4.52	4.23 ± 5.64	0.858
ALT (U/L)	32.97 ± 43.51	24.97 ± 24.49	0.191
AST (U/L)	24.72 ± 22.59	20.95 ± 10.93	0.233
γ-GT (U/L)	29.78 ± 27.68	24.48 ± 17.79	0.259
TC (mmol/L)	5.22 ± 1.10	4.79 ± 0.98	0.004**
TG (mmol/L)	1.42 ± 0.99	1.35 ± 0.97	0.631
LDL-c (mmol/L)	3.47 ± 1.05	2.98 ± 0.76	0.002**
HDL-c (mmol/L)	1.36 ± 0.42	1.32 ± 0.37	0.553
FFA (mmol/L)	0.50 ± 0.19	0.58 ± 0.23	0.082
Cr (μmol/L)	56.81 ± 7.07	55.94 ± 8.44	0.474
UA (μmol/L)	356.32 ± 87.44	336.76 ± 90.56	0.138
BUN (mmol/L)	4.53 ± 1.30	4.30 ± 1.10	0.283
eGFR (ml/min)	122.23 ± 9.57	121.83 ± 10.48	0.816
LH (IU/L)	12.16 ± 9.94	13.30 ± 9.55	0.411
FSH (IU/L)	6.22 ± 2.02	5.62 ± 1.92	0.031*
LH/FSH	1.93 ± 1.14	2.41 ± 1.39	0.014*
PRL (mIU/L)	346.67 ± 200.82	415.20 ± 288.36	0.089
E2 (pmol/L)	251.99 ± 222.41	285.48 ± 278.42	0.393
TT (nmol/L)	1.89 ± 0.74	2.34 ± 3.30	0.316
FT (pg/mL)	3.67 ± 1.74	4.52 ± 2.03	0.007**
AD (ng/mL)	4.78 ± 2.10	5.35 ± 2.01	0.064
DHEA-S (μg/dL)	275.57 ± 131.35	304.03 ± 147.16	0.194
SHBG (nmol/L)	40.27 ± 37.04	39.97 ± 34.58	0.955
AMH (ng/ml)	6.12 ± 4.59	7.99 ± 4.43	0.010*
Left OV (cm^3^)	8.00 ± 2.97	9.00 ± 3.88	0.071
Right OV (cm^3^)	10.91 ± 9.07	10.64 ± 5.20	0.833
Left FN (n)	23.21 ± 6.11	21.50 ± 8.37	0.078
Right FN (n)	24.25 ± 8.73	22.44 ± 8.67	0.158

Data are presented as mean ± SD. *P < 0.05; **P < 0.01, ***P < 0.001.

BMI, body mass index; WC, waist circumference; WHR, waist circumference to hip circumference ratio; HOMA-IR, homeostasis model assessment of insulin resistance; HbA1c, glycosylated hemoglobin A1; ALT, alanine aminotransferase; AST, aspartate aminotransferase; TC, total cholesterol; TG, triglyceride; HDL-c, high-density lipoprotein cholesterol; LDL-c, low-density lipoprotein cholesterol; Cr, creatinine; UA, uric acid; BUN, blood urea nitrogen; γ-GT, γ-glutamyl transpeptidase; eGFR, estimated glomerular filtration rate; LH, luteinizing hormone; FSH, follicle-stimulating hormone; PRL, prolactin; E2, estradiol e2; TT, total testosterone; FT, free testosterone; Androstenedione, AD; DHEA-S, dehydroepiandrosterone sulfate; SHBG, sex hormone-binding globulin; AMH, anti-Müllerian hormone; Left OV, left ovarian volume; Right OV, right ovarian volume; Left FN, left ovarian follicle number; Right FN, right ovarian follicle number.

### Changes in anthropometric, biochemical and reproductive variables

The mean time to achieve PCOS remission was 12.76 months, with a range of 3.00–49.50 months. At baseline, among the 55 patients, 17 (30.91%) were obese (BMI≥28 kg/m²), 13 (23.64%) were overweight (24≤BMI<28 kg/m²), 23 (41.82%) had a normal BMI (18.5≤BMI<24 kg/m²) and 2 (3.64%) were underweight (BMI < 18.5 kg/m²). There was a significant reduction in BMI (26.13 kg/m² to 24.00 kg/m², p<0.001), WC (88.10 cm to 82.75 cm, p<0.001) and WHR (0.87 to 0.85, p=0.007) among patients in the remission group after treatment. The number of menstrual cycles per year showed a significant increase after treatment compared with baseline (6.93 to 10.51, p<0.001). The levels of ALT decreased significantly from 33.18 U/L to 17.68 U/L (p=0.004), as did those of AST, which declined from 24.94 U/L to 18.39 U/L (p=0.034). The level of γ-GT decreased from 23.22 U/L to 15.62 U/L (p=0.043). Similarly, TC levels dropped from 5.22 mmol/L to 4.79 mmol/L (p=0.003), and LDL-c levels decreased from 3.48 mmol/L to 2.93 mmol/L (p<0.001). UA levels declined from 352.83 μmol/L to 299.28 μmol/L (p<0.001). There was a significant increase in E2, from 234.29 pmol/L to 335.63 pmol/L (p=0.042). Total testosterone levels also decreased significantly, from 1.89 nmol/L to 1.34 nmol/L (p<0.001). Additionally, significant decreases were observed in AD (5.01 ng/mL to 3.73 ng/mL, p=0.001) and AMH (5.96 ng/ml to 4.84 ng/ml, p=0.029). No significant differences were observed in HbA1c, HOMA-IR, TG, HDL-c, FFA, Cr, BUN, LH, FSH, PRL, FT, DHEA-S, or SHBG (**Table 2**).

**Table 2:** Demographic, serological, and ultrasonographic characteristics of the remission group: pre- and post-treatment comparisons.

Items	Pre-treatment	Post-treatment	P values
BMI (kg/m^2^)	26.13 ± 5.71	24.00 ± 4.74	<0.001***
WC (cm)	88.10 ± 12.35	82.75 ± 10.01	<0.001***
WHR	0.87 ± 0.07	0.85 ± 0.07	0.007**
Menstrual cycles (no./year)	6.93 ± 3.67	10.51 ± 1.24	<0.001***
HbA1c (%)	5.53 ± 0.76	5.40 ± 0.34	0.279
HOMA-IR	4.24 ± 4.72	2.74 ± 3.39	0.066
ALT (U/L)	33.18 ± 44.24	17.68 ± 14.52	0.004**
AST (U/L)	24.94 ± 23.21	18.39 ± 7.81	0.034*
γ-GT (U/L)	23.22 ± 16.32	15.62 ± 6.78	0.043*
TC (mmol/L)	5.22 ± 1.11	4.79 ± 0.74	0.003**
TG (mmol/L)	1.41 ± 1.00	1.18 ± 0.69	0.113
LDL cholesterol (mmol/L)	3.48 ± 1.06	2.93 ± 0.74	<0.001***
HDL cholesterol (mmol/L)	1.35 ± 0.42	1.35 ± 0.36	0.997
FFA (mmol/L)	0.48 ± 0.21	0.47 ± 0.31	0.955
Cr (μmol/L)	56.76 ± 6.84	55.49 ± 10.65	0.376
UA (μmol/L)	352.83 ± 86.93	299.28 ± 84.54	<0.001***
BUN (mmol/L)	4.56 ± 1.43	4.50 ± 1.34	0.805
eGFR (ml/min)	122.23 ± 9.26	119.78 ± 10.22	0.043*
LH (IU/L)	12.09 ± 9.84	10.63 ± 11.89	0.496
FSH (IU/L)	6.20 ± 1.99	5.56 ± 2.49	0.088
LH/FSH	1.92 ± 1.12	1.95 ± 1.17	0.863
PRL (mIU/L)	355.42 ± 202.67	421.02 ± 216.32	0.075
E2 (pmol/L)	234.29 ± 201.48	335.63 ± 302.27	0.042*
TT (nmol/L)	1.89 ± 0.76	1.34 ± 0.54	<0.001***
FT (pg/mL)	3.75 ± 1.80	3.42 ± 1.15	0.262
AD (ng/mL)	5.01 ± 2.13	3.73 ± 2.04	0.001*
DHEA-S (μg/dL)	281.96 ± 138.77	288.90 ± 170.66	0.720
SHBG (nmol/L)	39.87 ± 39.50	45.02 ± 43.48	0.285
AMH (ng/ml)	5.96 ± 4.94	4.84 ± 3.07	0.029*
Left OV (cm^3^)	7.97 ± 3.02	6.23 ± 1.84	0.001**
Right OV (cm^3^)	10.35 ± 8.28	7.03 ± 1.96	0.006**
Left FN (n)	23.23 ± 6.35	12.05 ± 3.99	<0.001***
Right FN (n)	23.59 ± 7.78	12.39 ± 3.85	<0.001***

Data were presented as mean ± SD. *P < 0.05; **P < 0.01; ***P < 0.001.

BMI, body mass index; WC, waist circumference; WHR, waist circumference to hip circumference ratio; HOMA-IR, homeostasis model assessment of insulin resistance; HbA1c, glycosylated hemoglobin A1; ALT, alanine aminotransferase; AST, aspartate aminotransferase; TC, total cholesterol; TG, triglyceride; HDL-c, high-density lipoprotein cholesterol; LDL-c, low-density lipoprotein cholesterol; Cr, creatinine; UA, uric acid; BUN, blood urea nitrogen; γ-GT, γ-glutamyl transpeptidase; eGFR, estimated glomerular filtration rate; LH, luteinizing hormone; FSH, follicle-stimulating hormone; PRL, prolactin; E2, estradiol e2; TT, total testosterone; FT, free testosterone; Androstenedione, AD; DHEA-S, dehydroepiandrosterone sulfate; SHBG, sex hormone-binding globulin; AMH, anti-Müllerian hormone; Left OV, left ovarian volume; Right OV, right ovarian volume; Left FN, left ovarian follicle number; Right FN, right ovarian follicle number.

### Changes in ultrasonographic characteristics

**Figure 2:**
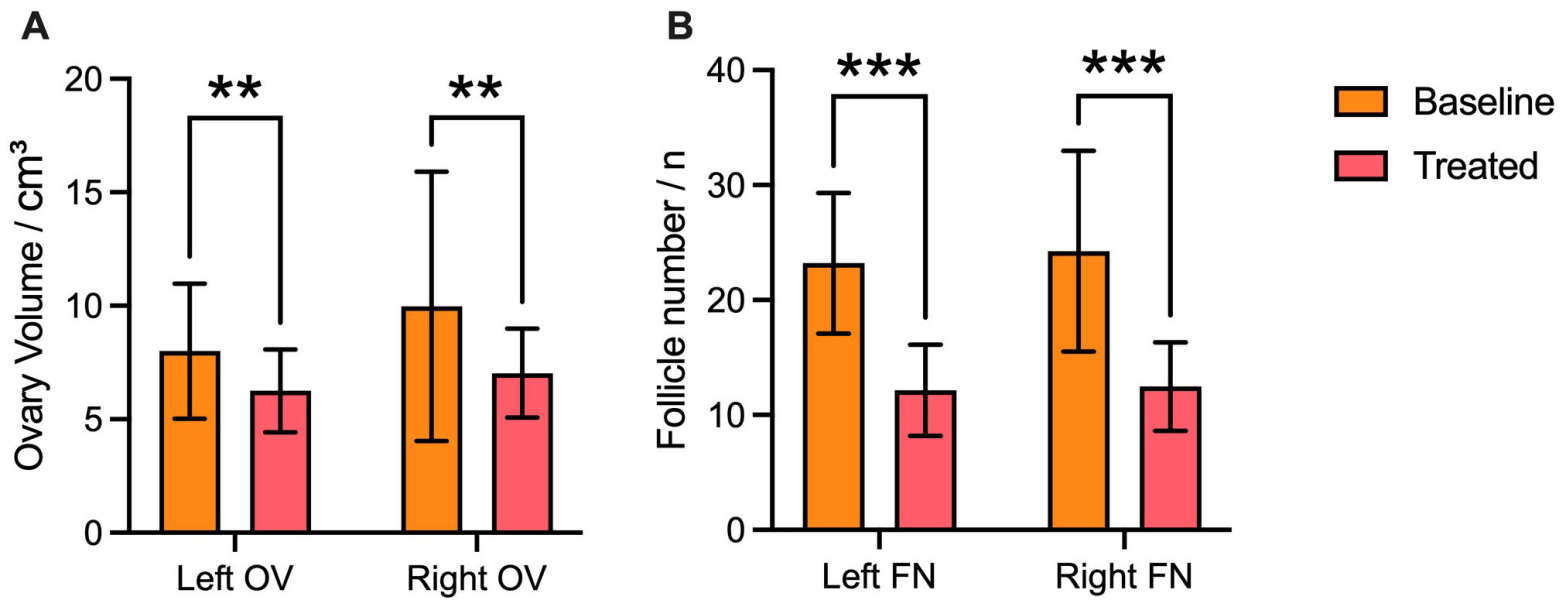
Comparison of ultrasonographic characteristics pre- and post-treatment in the remission group. **(A)** Comparison of left and right OV. **(B)** Comparison of left and right FN. Data are presented as means with error bars representing standard deviations. **P < 0.01; ***P < 0.001. OV, ovarian volume; FN, follicle number; baseline, pre-treatment data in remission group; treated, post-treatment data in remission group.

**Figure 3:**
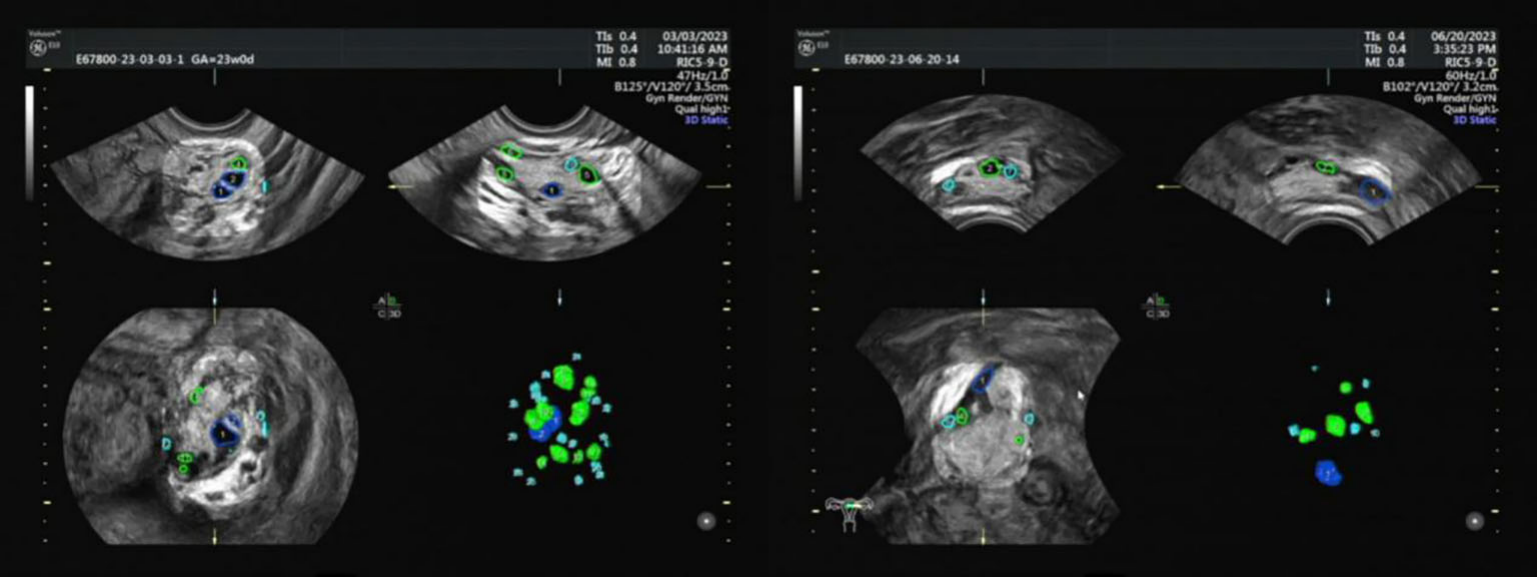
Comparison of ultrasonographic images before and after remission. These were ultrasonographic images of Patient No. 3 in the remission group. The left image depicted the polycystic ovarian morphology, while the right image showed the ovarian ultrasonographic characteristics after post-treatment remission. Follicles of varying sizes were marked in green and blue.

Following pharmacological intervention, ultrasonographic assessments revealed significant reductions in ovarian parameters (**Table 2**). The left ovarian volume decreased from 7.97 cm³ to 6.23 cm³ (p=0.001), while the right ovarian volume reduced from 10.35 cm³ to 7.03 cm³ (p=0.006) (**Figure 2A**). Additionally, the follicle number (FN) in both ovaries showed marked decreases: left ovary from 23.23 to 12.05 (p<0.001); right ovary from 23.59 to 12.39 (p<0.001) (**Figure 2B**). Transvaginal gynecologic ultrasound images of Patient No.3 in the remission group demonstrated changes in FNPO and OV before and after treatment (**Figure 3**).

### Medication strategies and BMI changes in patients with PCOS remission

**Figure 4:**
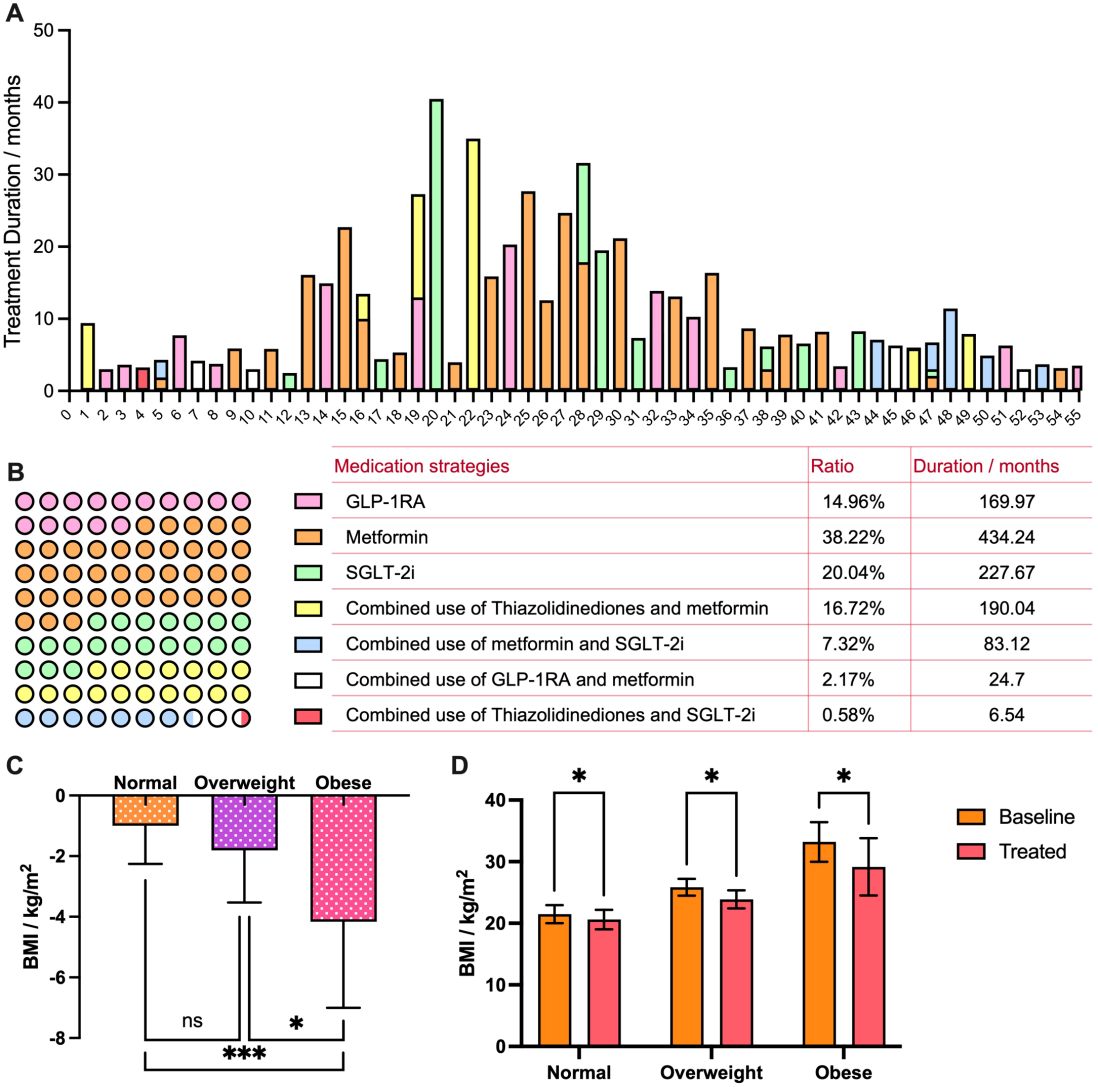
Medication strategies, treatment duration, and BMI changes in patients with PCOS remission. **(A)** Medication strategies and treatment duration in the remission group. **(B)** Proportional distribution of each medication strategy among patients achieving remission. **(C)** Comparison of pre- and post-treatment BMI changes across BMI-based subgroups. **(D)** Intra-subgroup analysis of BMI changes before and after treatment within each BMI stratum. Data are presented as means with error bars representing standard deviations. ns, not significant; *P < 0.05; ***P < 0.001. GLP-1RAs, glucagon-like peptide-1 receptor agonists; SGLT-2is, sodium-glucose linked transporter 2 inhibitors; TZDs, thiazolidinediones; baseline, pre-treatment data in remission group; treated, post-treatment data in remission group.

**Figure 4A** illustrated the treatment strategies employed for each patient within the remission group, accompanied by the duration ratios of these strategies. These strategies consisted of a variety of medications and combinations, including GLP-1RAs, metformin, SGLT2is and TZDs. Notably, metformin monotherapy accounted for 38.22%, SGLT2is monotherapy for 20.04% and GLP-1RAs monotherapy for 14.96% of all medication strategy durations in the remission group (**Figure 4B**). And TZDs were used in a substantial proportion of patients (17.30%) as part of the combination regimen.

As depicted in **Figure 4C**, BMI changes across normal-weight, overweight, and obese subgroups of patients with PCOS in clinical remission were analyzed. The obese subgroup showed a significantly greater reduction in BMI relative to both the normal-weight subgroup (P<0.001) and the overweight subgroup (P < 0.050), whereas no significant difference was observed between normal-weight and overweight subgroups. As shown in **Figure 4D**, within-subgroup analyses demonstrated a significant reduction in post-treatment BMI among participants stratified into normal-weight, overweight, and obese categories (all P < 0.050).

### Univariable and multivariable Cox proportional hazards regression analysis

Univariable and multivariable Cox proportional hazards regression was performed to identify independent factors associated with remission in patients with PCOS (**Table 3**). Univariable Cox proportional hazards regression (Model 1) identified several factors associated with PCOS remission, including BMI (HR = 1.054, 95% CI: 1.003–1.107, p=0.038), ALT (HR = 1.007, 95% CI: 1.000–1.013, p=0.041), LDL-c (HR = 1.823, 95% CI: 1.321–2.514, p < 0.001), AD (HR = 0.853, 95% CI: 0.741–0.982, p=0.027), and AMH (HR = 0.905, 95% CI: 0.827–0.991, p=0.031). Notably, no significant association was observed between medication use and PCOS remission in the univariable analysis (data not shown).

**Table 3:** Univariable and Multivariate Cox analyses of factors associated with the remission of PCOS.

Items	Model 1: HR (95% CI) P values	Model 2: HR (95% CI) P values	Model 3: HR (95% CI) P values
BMI (kg/m^2^)	1.054 (1.003, 1.107) p=0.038*	1.054 (1.001, 1.111) p=0.046*	1.062 (1.000, 1.128) p=0.048*
ALT (U/L)	1.007 (1.000, 1.013) p=0.041*	1.007 (1.001, 1.014) p=0.032*	1.011(1.002, 1.021) p=0.016*
LDL-c (mmol/L)	1.823 (1.321, 2.514) p<0.001***	1.953 (1.387, 2.748) p<0.001***	1.874(1.291, 2.722) p=0.001**
AD (ng/mL)	0.853 (0.741, 0.982) p=0.027*	0.880 (0.759, 1.019) p=0.088	0.910 (0.774, 1.070) p=0.252
AMH (ng/ml)	0.905 (0.827, 0.991) p=0.031*	0.902 (0.824, 0.987) p=0.025*	0.907 (0.819, 1.006) p=0.065

Data are presented as Hazard Ratios (HR) with 95% Confidence Intervals (CI).

Model 1: Unadjusted (crude) analysis.

Model 2: Adjusted for age (years) and menstrual cycles (no./year).

Model 3: Additionally adjusted for HbA1c,TG, Cr and mean OV based on Model 2.

*P < 0.05; **P < 0.01; ***P < 0.001.

HR, hazard ratio; CI, confidence interval; BMI, body mass index; ALT, alanine aminotransferase; LDL-c, low-density lipoprotein cholesterol; AD, androstenedione; AMH, anti-Müllerian hormone; HbA1c, glycated hemoglobin; TG, triglycerides; mean Cr, creatinine; OV, mean ovarian volume.

After adjusting for age and menstrual cycles per year (Model 2), BMI (HR = 1.054, 95% CI: 1.001–1.111, p=0.046), ALT (HR = 1.007, 95% CI: 1.001–1.014, p=0.032), LDL-c (HR = 1.953, 95% CI: 1.387–2.748, p < 0.001), and AMH (HR = 0.902, 95% CI: 0.824–0.987, p=0.025) remained significant, while AD showed a borderline association (HR = 0.880, 95% CI: 0.759–1.019, p=0.088). In the Model 3, which additionally accounted for HbA1c, TG, Cr, and mean OV based on model 2, BMI (HR = 1.062, 95% CI: 1.000–1.128, p=0.048), ALT (HR = 1.011, 95% CI: 1.002–1.021, p=0.016), and LDL-c (HR = 1.874, 95% CI: 1.291–2.722, p=0.001) remained independent predictors of remission. However, the associations of AD and AMH with remission did not show statistical significance in Model 3 (both p > 0.05).

## Discussion

PCOS is a metabolic disease characterized by both endocrine and reproductive features, as well as an increased prevalence of hyperinsulinemia and insulin resistance (24). Given its elevated risk of cardiovascular disease and metabolic syndrome (5), achieving PCOS remission has emerged as a critical clinical priority. PCOM—a feature present in approximately 90% of patients with PCOS—is typically assessed using both 2D and 3D ultrasound imaging. Notably, this work incorporated 3D ovarian ultrasonography to assess the resolution of PCOM, which may supply objective morphological indicators to aid prognostic evaluation and inform clinical management strategies for individuals with PCOS.

In the present study, 55 out of 468 patients with PCOS (11.75%) achieved PCOS remission, as evidenced by restored ovarian morphology, normalized testosterone concentrations, and regular menstrual cycles. The mean time to achieve PCOS remission was 12.76 months. Although the remission group and non-remission group exhibited comparable age, BMI, HbA1c levels, HOMA-IR, liver and renal function, the remission group displayed significantly higher WHR, TC and LDL-c levels along with lower FT and AMH levels. In the remission group, BMI, WC, and WHR all showed significant reductions after treatment, indicating that the intervention effectively promoted weight loss and reduced abdominal fat in PCOS patients. This finding aligned with previous research suggesting that a 5-10% weight loss can improve PCOS symptoms (24–27). It was also consistent with prior studies indicating that patients with abdominal obesity-–often associated with insulin resistance and hyperinsulinemia-–are more likely to experience improvements with medication or surgical intervention (24, 28–30). Several studies have also established a correlation between weight reduction and the restoration of menstrual regularity and ovulation (31, 32). Notably, normal weight patients accounted for the largest proportion of the remission group (41.82%) and BMI was decreased post-treatment in normal-weight subgroups, suggesting that metabolic modulation can help alleviate PCOS with normal weight (33, 34).

Following treatment, the remission group exhibited significant reductions in AST, ALT, TC, and LDL-c levels. Elevated AST/ALT levels exacerbate PCOS pathogenesis through the SHBG-free testosterone axis and the inflammatory-insulin resistance pathway, while their normalization directly ameliorates hyperandrogenemia and promotes ovulation restoration (35). The significant negative correlation between TC levels and remission suggests that lower baseline TC concentrations and subsequent improvements in lipid profiles may confer favorable effects. Improvements in lipid profiles may enhance the follicular microenvironment and ovulation by reducing ovarian oxidative stress and androgen synthesis (36, 37). Hyperinsulinemia stimulates ovarian theca cells to increase androgen production by activating the P450c17α enzyme and suppresses hepatic synthesis of sex hormone-binding globulin (SHBG), thereby elevating the bioavailability of free testosterone in the circulation (38–40). Even partial improvements in insulin resistance can suppress androgen overproduction and promote PCOS remission (41).

The remission group demonstrated significant reductions in bilateral ovarian volume and antral follicle count, accompanied by a decline in AMH levels. These findings highlight the potential reversibility of pathological ovarian alterations and the capacity for morphological restoration after standardized pharmacological intervention in PCOS patients. This morphological improvement may be explained by metabolic regulation. Lee JE et al. have reported that total antral follicle count was found to be related to fasting insulin resistance-related parameters in women with PCOS (42). Agents for improving metabolism may improve ovarian follicular cell architecture by inducing antioxidant and anti-inflammatory systems via SIRT1 and AMPK activation in PCOS (43). In our study, weight loss and improved glucose-lipid metabolism could restore ovulation, lower androgens, and AMH. Consequently, ameliorated ovarian sex hormones enhance metabolic indicators, forming a benign cycle. This reciprocal interaction might reinforce metabolic-hormonal stability, thereby driving recovery of polycystic ovarian morphology and enhance overall reproductive-metabolic health in individuals with PCOS.

In the remission group, the therapeutic strategies employed included a range of medications and combination therapies, such as GLP-1RAs, metformin, SGLT2is, and TZDs. Several studies have shown that monotherapy or combination therapy with these agents can effectively reduce testosterone levels, restore menstrual regularity, ameliorate insulin resistance, and promote weight loss (27, 44–47). The shared mechanisms of these medications include improving insulin resistance, reducing inflammation, alleviating oxidative stress, and modulating energy metabolism (6, 44, 48–53). Furthermore, mechanistic studies have indicated that these medication strategies achieve reductions in androgen synthesis either by activating AMPK or by directly suppressing androgen production through downregulating the expression of CYP17A1 and 3β-HSD in theca cells (25, 51).

Univariable Cox regression analysis indicated that higher BMI, ALT, and LDL-c, as well as lower AD and AMH, were associated with a higher hazard of PCOS remission. Prior research reported that high preconception AD confers an elevated miscarriage risk (54). Similarly, AMH displays inverse correlations with clinical pregnancy rate, live birth rate, cumulative live birth rate, and ovulatory restoration following bariatric metabolic surgery (55, 56). Intriguingly, although BMI, ALT and LDL-c remained robustly predictive of remission after adjusting for multiple confounding variables, their prognostic direction was counterintuitive. Conventionally, elevated metabolic parameters correspond to aggravated PCOS pathophysiology and are assumed to hinder disease recovery (57–60). However, this apparent discrepancy may be partially attributable to the distinctive features of our study cohort. Unlike participants in most previous studies who received no standardized metabolic intervention (58–60), all individuals in the present cohort underwent unified metabolic-correcting pharmacotherapy. However, no significant association between medication use and PCOS remission was identified in the Cox regression analyses. We hypothesize that this lack of statistical significance may be attributed to the widespread administration of metabolic-modifying agents throughout the entire cohort, which diminished intergroup heterogeneity and masked the independent therapeutic effects of these medications on disease remission.

## Limitations

This study was limited by the relatively small sample size of the remission group and the substantial attrition from the initial cohort to the final analytical sample, which may introduce selection bias and restrict the generalizability of the reported PCOS remission rate. As a retrospective clinical study, it relies on historical data, potentially leading to issues with data completeness and quality and introducing reporting bias. Missing metabolic biomarker measurements for a subset of individuals in the non-remission group prevented additional analyses of longitudinal links between dynamic metabolic shifts and subsequent PCOS remission status. Additionally, the lack of an objective and standardized measure for assessing patients’ adherence to lifestyle interventions may introduce detection bias. Overall, these limitations indicate that further research with larger, more representative samples is required to validate and extend the results of this study.

## Strengths

This study demonstrated that 11.75% of women with PCOS achieved disease remission following metabolic-modulating therapy, as evidenced by restored ovarian morphology, normalized testosterone concentrations, and regular menstrual cycles. The marked reductions in both ovarian volume and follicle count underscore the key role of targeting PCOM in PCOS management. Remission of PCOM represents a central indicator of pathological improvement in PCOS, which is critical for optimizing fertility outcomes, reducing long-term chronic disease-related complications, and avoiding treatments that only alleviate symptoms without addressing the underlying pathophysiology.

## Conclusion

Our observations offer preliminary evidence suggesting that patients with PCOS may attain restoration of ovarian morphology, alongside favorable shifts in reproductive and metabolic profiles, following interventions aimed at ameliorating underlying metabolic dysfunction. The findings imply that prioritizing metabolic correction represents a promising therapeutic strategy to reverse ovarian structural aberrations and alleviate the broad range of PCOS-associated clinical phenotypes.

## Data Availability

The datasets generated and analyzed during the current study are not publicly available due to the need to protect patient confidentiality, in accordance with the data security policies of Shanghai Tenth People's Hospital, but are available from the corresponding author upon reasonable request.
